# Comparative effect of deliberate hypotensive anesthesia using nitroglycerine vs. phentolamine on event related potentials and cognitive functions in patients undergoing septoplasty: a randomized controlled trial

**DOI:** 10.1186/s12871-023-02096-y

**Published:** 2023-05-03

**Authors:** Wael Fathy, Mona Hussein, Rehab Magdy, Hanan H Soliman, Hatem Elmoutaz, Alaa A Meshref, Reem M Sabry, Marwa A Elgaly, Mohammed Fawaz, Dina Y Kassim

**Affiliations:** 1 Department of Anesthesia, Surgical ICU and Pain management, Beni-Suef University, Beni-Suef, Egypt; 2 Department of Anaesthesia, Surgical ICU and Pain management, Beni-Suef University, Salah Salem Street, Beni-Suef, Egypt; 3 Department of Neurology, Beni-Suef University, Beni-Suef, Egypt; 4 Department of Neurology, Cairo University, Cairo, Egypt; 5 Neuro diagnostic research center, Beni-Suef University, Beni-Suef, Egypt; 6 Department of Otorhinolaryngology, Beni-Suef University, Beni-Suef, Egypt

**Keywords:** Deliberate hypotensive anesthesia, Nitroglycerine, Phentolamine, PALT, BVRT, P300

## Abstract

**Background:**

Postoperative cognitive dysfunction is a noteworthy complication of deliberate hypotensive anesthesia. The aim of this work was to compare the effect of deliberate hypotensive anesthesia using nitroglycerine versus phentolamine on event-related potentials and cognitive function in patients undergoing septoplasty surgery.

**Methods:**

This prospective randomized controlled trial was conducted on 80 patients indicated for septoplasty under general anesthesia; 40 patients received intra-operative Nitroglycerine and 40 patients received intra-operative Phentolamine. Cognitive assessment (using Paired Associate Learning test (PALT) and Benton Visual Retention test (BVRT)) and P300 recording were done for all included patients pre-operatively and one week postoperatively.

**Results:**

The scores of PALT and Benton BVRT significantly declined one week following surgery in both Nitroglycerine and Phentolamine groups. There was no statistically significant difference between Nitroglycerine and Phentolamine groups in the postoperative decline in either PALT or BVRT (P-value = 0.342, 0.662 respectively). The values of P300 latency showed a significant delay one week following surgery in both Nitroglycerine and Phentolamine groups (P-value ≤ 0.001, 0.001), but in Nitroglycerine group, the delay is significantly higher than in Phentolamine group (P-value = 0.003). The values of P300 amplitude significantly decreased one week following surgery in both Nitroglycerine and Phentolamine groups (P-value ≤ 0.001, 0.001), but there was no statistically significant difference between Nitroglycerine and Phentolamine groups (P-value = 0.099).

**Conclusion:**

Phentolamine is preferred over nitroglycerin in deliberate hypotensive anesthesia because it has less harmful effect on cognitive function than nitroglycerin.

## Introduction

Postoperative cognitive dysfunction (POCD) has gained much attention in the past years. Despite the great strides in anesthesia and surgical techniques over the past decades, POCD is still highly prevalent [1]. The exact pathophysiology of POCD remains unknown. However, several risk factors of POCD were determined, such as old age, preoperative cognitive impairment, and the perioperative events related to the surgery itself [1, 2]. Whether or not and to what extent anesthetic drugs contribute to POCD remains unclear [3].

Deliberate hypotensive anesthesia is necessary during maxillofacial and septoplasty surgery for drying surgical field, ease in operation procedure and shortening of the duration of surgery. However, deliberate hypotensive anesthesia is associated with increased risk of impaired perfusion to important organs, such as the brain [4]. Many hypotensive drugs with different mechanisms and duration of action were investigated for being implemented in causing POCD [5, 6].

The mechanism of the hypotensive action of phentolamine is mediated through its nonselective α-adrenergic antagonist activity, resulting in a decrease in peripheral vascular resistance and vasodilatation. [7]. Some hypotensive drugs, such as nitroglycerin, can produce an increase in intracranial pressure (ICP) in addition to its direct vasodilator effect, which is mediated by nitric oxide. Consequently, we hypothesize that nitroglycerin may have a more deleterious effect on cognitive function than other hypotensive drugs [8, 9].

Neuropsychological tools are classically used to investigate POCD. The optimal cognitive battery is one that includes highly sensitive different tests, each targeting a specific cognitive area [10]. Since memory is the most vulnerable domain in POCD [1], we chose to test both types of memory in this study (verbal & visual) by two different tests. Calls for better diagnosis of POCD have emphasized the integration of other tools. The utility of Event-related potentials (ERPs) is promising in this area [11, 12]. They can examine a range of electrophysiological components in a short period with no subjective variability related to the examiners [13].

This work aimed to compare the effect of deliberate hypotensive anesthesia using nitroglycerine versus phentolamine on ERPs and cognitive function in patients undergoing septoplasty surgery to select the most appropriate agent to induce hypotension.

## Methods

### Study design and population

This prospective randomized controlled trial was conducted on 80 patients indicated for septoplasty under general anesthesia. Patients were randomly assigned into one of two groups; the first group (40 patients) received intra-operative Nitroglycerine (Nitroglycerine group) and the second group (40 patients) received intra-operative Phentolamine (Phentolamine group). Randomization was carried out using a closed opaque envelope technique where the physician picked up a sealed envelope containing a card having the name of the group to which the patient was randomly selected. Whatever group was written on the card, the patient was scheduled to it.

The patients were recruited from Otorhinolaryngology surgery department, Beni-Suef University Hospital, from January 2020 to May 2021. The study was registered in ClinicalTrials.gov on 1/10/2019, and this is the identification number NCT04110808.

### Eligibility criteria

The study included American Society of Anesthesiologists I, II (ASA I, II) patients who were candidate for septoplasty. The age range was between 20 and 50 years. The following patients were excluded from the study: patients who developed intraoperative hypotension with mean arterial blood pressure (MAP) less than 60 mmHg, patients with a history of neurodegenerative disorder, patients with a concomitant medical or metabolic illness known to affect cognition, patients with visual or auditory dysfunction that might affect their ability to respond to cognitive assessment, and patients who were allergic to any of the anesthetic or hypotensive drugs used in the study.

### Cognitive assessment

Cognitive assessment was done for all included patients pre-operatively and one week postoperatively by an expert neurologist who was blinded to the used intraoperative drugs, using the following psychometric tests:

1. **Paired Associate Learning test (PALT)** [14] was used to assess auditory verbal memory. In this test, the examiner says 6 semantically related pairs of words and 4 semantically unrelated pairs. After a few minutes, the examiner said the first word of each pair to the patient, and the patient was asked to recall the other word. The test must be performed three times. Each correct compatible pair takes a score 0.5, while each correct incompatible pair takes a score 1. The range of the total score was between 0 and 21.
2. ***Benton Visual Retention test (BVRT)*** [15]: It was used to assess visual memory. In this test, 10 cards are shown to the patient, the first two cards contain one large figure, and the other 8 cards contain two large figures and a smaller peripheral figure. Each card is shown to the patient for few seconds then removed. The patient is instructed to recall the figures in each card and draw them it on a blank sheet of paper. Each correct figure is given a score 1. The range of the total score was between 0 and 26.

### Neurophysiological assessment

P300 recording was done for all included patients pre-operatively and one week postoperatively by an expert neurophysiologist who was blinded to the intraoperative drugs used. The used device was Galileo Series preamplifiers acquisition system (EBN, Florence, Italy). Bioelectrical activity of the brain was recorded by using gold electrodes and a conductive EEG paste (Elefix). Three active electrodes were positioned at the central line of the scalp frontally (Fz), centrally (Cz), and parietally (Pz) according to the international 10–20 system. While reference electrodes were placed on the earlobes, the ground electrode was positioned at the forehead. Impedance of all electrodes in all recordings was under 5 kΩ. The patients were instructed to keep their eyes closed and avoid head movements in order to reduce eye and muscle artifacts during P300 recording. All participants underwent auditory oddball paradigm task testing. They were instructed to listen to the series of tones with closed eyes. The auditory stimuli were pure tones presented binaurally at random intervals ranging between 3 and 4 s. All tones were 100 ms in duration with a rise–fall time of 10ms and were adjusted in intensity to a 70-dB sound pressure level. The pitches of frequent (standard) and infrequent (target) tones were 1000 and 3000 Hz, respectively, with a total number of 200 tones and a presentation probability for the infrequent tones of (0.2). The patients were asked to identify the infrequent tones in one task by raising the right index finger of the dominant hand in response to the infrequent tones as targets during 15-minute session.

The ERP data were averaged with the sweep beginning 100 ms before the stimuli and lasting until 900 ms after stimulus onset in a common average montage containing 3 channels of midline electrode locations Fz, Cz, and Pz according to the international 10–20 system of EEG electrode placement. The P300 latency was identified as the largest positive peak at range of 250–700 ms occurring after the N1, P2, and N2 ERP components obtained from the ‘‘target’’ stimulus presentation. The peak amplitudes were evaluated as the differences between the P300 peak and the mean baseline automatically calculated by a computer [15].

### Anesthetic technique

At the operating room, standard patient monitoring was established (electrocardiography, pulse oximetry, end-tidal carbon dioxide (ET CO2 ), and noninvasive arterial blood pressure monitoring). Two intravenous cannulas were placed; 18 and 20 gauge.

Induction of anesthesia was done by injecting 2 mg/kg propofol, 2 µg/kg fentanyl & 0.5 mg/kg atracurium. The patients were ventilated via face mask with 100% oxygen at a rate of 6 L/min and isoflurane 1.2%. After few minutes, the patients were intubated using a cuffed oral tube. Maintenance of anesthesia was done using isoflurane 1.2% in a mixture of 70% oxygen and 30% air. Muscle relaxation was continued with atracurium 0.1 mg/kg every 20 min. All patients were mechanically ventilated to maintain ET CO2 between 35 and 40 mmHg. An intra-arterial canula was inserted in the radial artery for invasive arterial blood pressure monitoring.

The patients were randomly allocated to one of the two groups; either Phentolamine or Nitroglycerine groups. In Phentolamine group (40 patients), the patients received deliberate hypotensive anesthesia with phentolamine infusion via syringe pump by adding 20 mg (2ml) of Phentolamine to 48 ml of normal saline, making it to a final concentration of 0.4 mg/ml at the rate of 0.1-2 mg/min according to the patients desired target blood pressure. In Nitroglycerine group (40 patients), the patients received deliberate hypotensive anesthesia with nitroglycerine infusion via syringe pump by adding 10 mg (10ml) of Nitroglycerin to 40ml of normal saline, making it to a final concentration of 200 µg/ml at the rate of 0.5–10 µg/kg/min according to the patients desired target blood pressure.

MAP was gradually reduced in both groups to maintain a target MAP of 60–70 mmHg and systolic blood pressure ≥ 85 mmHg. Patients who developed hypotension (MAP less than 55 mmHg) were managed by discontinuation of the hypotensive drug and administration of intravenous fluid. Ephedrine (10 mg) was given IV when the MAP did not improve, and these patients were excluded from the study.

Infusion of the hypotensive agent was stopped 10 min before the anticipated end of surgery. Any residual neuromuscular block was antagonized with neostigmine 0.04 mg/kg and atropine 0.02 mg/kg IV. The patients were extubated after fulfilling the criteria of recovery and transferred to the recovery room.

### Sampling

Because our study was the first study to compare the effect of deliberate hypotensive anesthesia using nitroglycerine versus phentolamine on P300, we calculated the sample size based on the results of a pilot study we performed before starting our study. The sample size calculation was done using G*Power version 3.1.9.7 Software. The probability of type I error (α) was 5%, effect size = 0.821, df = 78, critical t = 1.99, noncentrality parameter λ = 3.673, A total sample size of 40 patients in each group was required to achieve a statistical power (1–β) 95%.

### Statistical analysis

IBM SPSS (Statistical Package of Social Science) Version 25 was used to analyze the data. Categorical variables such as sex were expressed as numbers and percentages. Quantitative variables such as age, years of education, PALT, BVRT, P300 latency, and amplitude were expressed as the median and interquartile range (IQR). Chi-squared test was used for comparison between Nitroglycerine and Phentolamine groups in sex, whereas Mann-Whitney test was used for comparison between Nitroglycerine and Phentolamine groups in age, years of education, PALT, BVRT, P300 latency, and amplitude. Wilcoxon test was used to compare pre and post-operative PALT, BVRT, P300 latency, and amplitude in each of Nitroglycerine and Phentolamine groups. A mixed ANOVA test was used to compare pre and post-operative PALT, BVRT, P300 latency, and amplitude in Nitroglycerine versus Phentolamine groups. P-value ≤ 0.05 was considered statistically significant. All tests were two-tailed.

## Results

### Demographics, preoperative psychometric tests, and P300 latency and amplitude

**Fig. 1:**
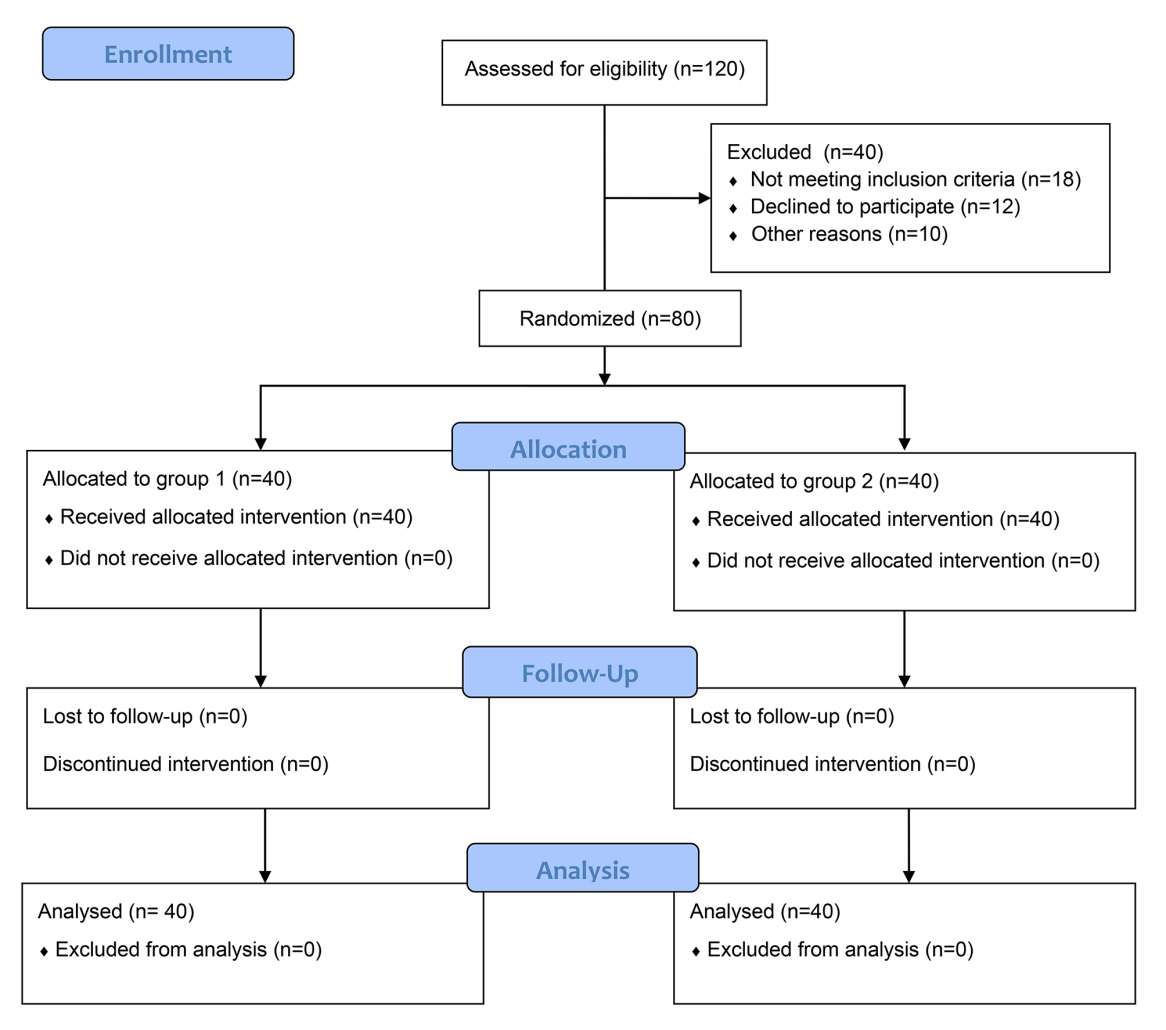
CONSORT 2010 Flow Diagram

This prospective randomized controlled trial was conducted on 80 patients indicated for septoplasty under general anesthesia. Forty patients received intra-operative Nitroglycerine (Nitroglycerine group), and 40 patients received intra-operative Phentolamine (Phentolamine group). Consort flow diagram is illustrated in Fig. (1). The median value for age in the Nitroglycerine group was 29 years with IQR (25.25-35) years, and in the Phentolamine group was 33.5 years with IQR (27–38) years. 20 (50%) patients in the Nitroglycerine group were males, and 20 (50%) were females. Whereas 25 (62.5%) patients in the Phentolamine group were males and 15 (37.5%) were females. There was no statistically significant difference between both groups in either age or sex (P-value = 0.193, 0.26 respectively) (Table 1). The median values and IQR for years of education, preoperative PALT, BVRT, P300 latency, and amplitude were demonstrated in Table (1). There was no statistically significant difference between Nitroglycerine and Phentolamine groups in these variables.

**Table 1:** Demographics, pre-operative psychometric tests, and pre-operative P300 latency and amplitude in patients in Nitroglycerine and Phentolamine groups

		Nitroglycerine group (n = 40)	Phentolamine group (n = 40)	P- value
Age [Median (IQR)]		29 (25.25-35)	33.5 (27–38)	0.193
Sex	Males [N (%)]	20 (50%)	25 (62.5%)	0.26
	Females [N (%)]	20 (50%)	15 (37.5%)	
Years of education [Median (IQR)]		12 (9–16)	14 (12–14)	0.8
Pre-operative PALT [Median (IQR)]		13.5 (11.5-15.88)	14.5 (12.5–15.5)	0.232
Pre-operative BVRT [Median (IQR)]		15 (11.25-17)	14 (12–17)	0.706
Pre-operative P300 latency [Median (IQR)]		322.7 (288.52- 344.86)	321.86 (309.53- 346.76)	0.535
Pre-operative P300 amplitude [Median (IQR)]		4.47 (2.98–6.41)	3.7 (2.4–4.5)	0.128

Values are expressed as median (Q1-Q3), number (%)

PALT: Paired Associate Learning Test, BVRT: Benton Visual Retention Test

Mann-Whitney test was used for comparison between quantitative variables, Chi-squared test was used for comparison between categorical variables, P-value > 0.05 is considered in-significant

### Effect of nitroglycerine versus phentolamine on the scores of psychometric tests

The scores of PALT significantly declined one week following surgery in both Nitroglycerine and Phentolamine groups (P-value = 0.034, 0.018 respectively). Also, the scores of BVRT significantly declined one week following surgery in both Nitroglycerine and Phentolamine groups (P-value = 0.013, 0.025 respectively). There was no statistically significant difference between Nitroglycerine and Phentolamine groups in the postoperative decline in either PALT or BVRT (P-value = 0.342, 0.662 respectively) (Table 2).

**Table 2:** Pre and post-operative psychometric tests in patients in Nitroglycerine and Phentolamine groups

Psychometric tests		Preoperative assessment [Median (IQR)]	Postoperative assessment [Median (IQR)]	P- value	P- value between groups
PALT	Nitroglycerine group	13.5 (11.5-15.88)	13 (11.25-15)	0.034*	0.342
	Phentolamine group	14.5 (12.5–15.5)	13 (12.25-15)	0.018*	
BVRT	Nitroglycerine group	15 (11.25-17)	13.5 (11- 16.75)	0.013*	0.662
	Phentolamine group	14 (12–17)	13 (11–16)	0.025*	

Values are expressed as median (Q1-Q3)

PALT: Paired Associate Learning Test, BVRT: Benton Visual Retention Test

Wilcoxon test was used for comparison between pre and post-operative quantitative variables, Mixed ANOVA test was used for comparing pre and post-operative quantitative variables in the two groups,

*P-value ≤ 0.05 is considered significant

There were no statistically significant differences between Nitroglycerine and Phentolamine groups in the percent of change in either PALT or BVRT (P-value = 0.689, 0.675 respectively) (Table 3).

**Table 3:** Percent of change in PALT and BVRT in both Nitroglycerine and Phentolamine groups

	Nitroglycerine group (n = 40)	Phentolamine group (n = 40)	P- value
Percent of change in PALT [Median (IQR)]	-3.85% (-10.83%-5.41%)	-7.96% (-12.2%- 6.16%)	0.689
Percent of change in BVRT [Median (IQR)]	-6.07% (-11.6% -8.33%)	0% (-16.67%- 8.33%)	0.675

Values are expressed as median (Q1-Q3)

PALT: Paired Associate Learning Test, BVRT: Benton Visual Retention Test

Mann-Whitney test was used for comparison between Nitroglycerine and Phentolamine groups

P-value > 0.05 is considered in-significant

### Effect of nitroglycerine versus phentolamine on P300 latency and amplitude

The values of P300 latency showed a significant delay one week following surgery in both Nitroglycerine and Phentolamine groups (P-value ≤ 0.001, 0.001), but in Nitroglycerine group, the delay is significantly higher than in Phentolamine group (P-value = 0.003). The values of P300 amplitude significantly decreased one week following surgery in both Nitroglycerine and Phentolamine groups (P-value ≤ 0.001, 0.001). There was no statistically significant difference between Nitroglycerine and Phentolamine groups in the postoperative decrease in P300 amplitude (P-value = 0.099) (Table 4).

**Table 4:** Pre and post-operative P300 latency and amplitude, in patients in Nitroglycerine and Phentolamine groups

P300		Preoperative assessment [Median (IQR)]	Postoperative assessment [Median (IQR)]	P- value	P- value between groups
Latency	Nitroglycerine group	322.7 (288.52- 344.86)	476.035 (430.135- 575.088)	< 0.001*	0.003*
	Phentolamine group	321.86 (309.53- 346.76)	413.77 (363.065–471.63)	< 0.001*	
Amplitude	Nitroglycerine group	4.47 (2.98–6.41)	1.93 (1.22–2.76)	< 0.001*	0.099
	Phentolamine group	3.7 (2.4–4.5)	1.28 (1.01–2.145)	< 0.001*	

Values are expressed as median (Q1-Q3)

Wilcoxon test was used for comparison between pre and post-operative quantitative variables, Mixed ANOVA test was used for comparing pre and post-operative quantitative variables in the two groups,

*P-value ≤ 0.05 is considered significant

The percent of change in P300 latency was significantly higher in Nitroglycerine group in comparison to Phentolamine group (P-value ≤ 0.001), but there was no statistically significant difference between Nitroglycerine and Phentolamine groups in the percent of change in P300 amplitude (P-value = 0.482) (Table 5).

**Table 5:** Percent of change in P300 latency and amplitude in both Nitroglycerine and Phentolamine groups

	Nitroglycerine group (n = 40)	Phentolamine group (n = 40)	P- value
Percent of change in P300 latency [Median (IQR)]	53.17% (33.96 − 80.22%)	23.22% (10.55 -51%)	≤ 0.001*
Percent of change in P300 amplitude [Median (IQR)]	-55.53% (-66.23% -37.36%)	-38.82% (-69.73%- 26.88%)	0.482

Values are expressed as median (Q1-Q3)

Mann-Whitney test was used for comparison between Nitroglycerine and Phentolamine groups

*P-value ≤ 0.05 is considered significant

## Discussion

Since deliberate hypotensive anesthesia is appropriate for patients who will undergo certain surgical procedures, including septoplasty surgery[4], a comparison of the effect of various hypotensive anesthetic agents with different mechanisms of action on patients’ cognitive function is a topic worthy of study. To our knowledge, this is the first study comparing the effect of nitroglycerine versus phentolamine use on postoperative cognitive function and event-related potentials.

In the presence of contradictions in the results of previous studies, whether or not hypotensive anesthesia causes POCD [16–19], we demonstrated in this study that both drugs caused such impairment, both in terms of cognitive tests and event-related potentials. The lack of a standardized and comprehensive set of cognitive batteries to determine the diagnosis of POCD may explain this considerable discrepancy between studies [10].

It is well-established that temporal lobe structures, especially the CA1 region of the hippocampus, are one of the most susceptible brain regions to cerebral hypoperfusion [20]. Indeed, the scores of the two neuropsychological tests significantly declined one week following surgery in both nitroglycerine and phentolamine groups in this study. Regardless of the different vasodilator mechanisms of each drug, they eventually lead to cerebral hypoperfusion.

Noteworthy, the results of neuropsychological tests might be confounded by some participant-related factors, including educational level, emotional state, and visual/auditory functions [21]. This is a key point in the interpretation of results of neuropsychological tests, requiring orientation towards other objective means.

As far as we know, this study is the first to investigate the effect of deliberate hypotensive anesthesia on Event-related potentials. Brain regions, such as the frontal cortex and the hippocampus, generate the P300 wave [22], which are structures usually affected by cerebral hypoperfusion [20]. Accordingly, this study showed that P300 latency and amplitude values were significantly delayed and decreased, respectively, in both the nitroglycerin and phentolamine groups.

Information processing speed is another cognitive domain commonly affected in POCD other than memory [1]. Although we did not use a cognitive test to assess the information processing speed in this study, P300 latency serves as an accurate indicator of information processing speed compared to P300 amplitude [23]. Since phentolamine outperformed nitroglycerin in this study, where the delay in the P300 latency was significantly higher in the latter than in the former group, we can say that the effect of phentolamine on the information processing speed is less than that of nitroglycerin.

Cerebral autoregulation is a compensatory mechanism that maintains cerebral blood flow constant despite a change in cerebral perfusion pressure (CPP) due to the effect of hypotensive agents on systemic vascular resistance [24]. Therefore, these significant declines in postoperative cognitive and neurophysiological tests are not due to the systemic hypotensive effects of nitroglycerin or phentolamine, as we used deliberate hypotension within the limits of mean blood pressure that allow constant cerebral blood flow.

It has to be mentioned that the reported POCD in our study may be attributed to the use of general anesthetic drugs. There is strong evidence suggesting the implication of general anesthetics in developing POCD. General anesthetics such as propofol and isoflurane were reported to cause neurodegeneration, neuroapoptosis, caspase activation, and B- amyloid protein accumulation, leading to POCD [25, 26].

Previous studies claimed that nitroglycerin causes an increase in ICP [9], so the CPP can be more affected with nitroglycerin than with phentolamine use. The CPP, which represents the difference between MAP and ICP, is the net pressure gradient that maintains oxygen delivery to cerebral tissue. This may explain why phentolamine outperformed nitroglycerin in the current study.

Whereas nitroglycerin is used broadly in multiple clinical practices in neurological and stroke intensive care units, our study implores limiting its use in anesthesia in neurologically affected patients, especially old age patients with cerebrovascular insufficiency giving chances to other hypotensive drugs in those patients.

Lastly, it should be noted that the relatively small sample size and cognitive follow-up for a relatively short period are the most critical limitations of the current study. Further studies with larger sample sizes and more extended follow-up periods are warranted.

## Conclusion

Phentolamine is preferred over nitroglycerin in hypotensive anesthesia for better postoperative cognitive outcomes in patients scheduled to undergo septoplasty.

## Data Availability

Authors report that the datasets used and/or analyzed during the current study are available from the corresponding author on reasonable request.

## Declarations

Authors report that the content has not been published or submitted for publication elsewhere.
